# Combined impacts of habitat degradation and cyclones on a community of small mammals

**DOI:** 10.1038/s41598-025-00740-w

**Published:** 2025-05-14

**Authors:** Veronarindra Ramananjato, Tanjoniaina H. N. P. Rabarijaonina, Tsinjo S. A. Andriatiavina, Finaritra Randimbiarison, Onja H. Razafindratsima

**Affiliations:** 1 Department of Integrative Biology, University of California Berkeley, Valley Life Science Building, Berkeley, CA 94720 USA; 2 Mention Zoologie et Biodiversité Animale, Faculté Des Sciences, Université d’Antananarivo, BP 906, 101 Antananarivo, Madagascar

**Keywords:** Body mass fluctuation, Climate change, Intermediate disturbance hypothesis, Population dynamics, Population ecology, Species resilience, Ecology, Systems biology, Zoology

## Abstract

We determined the combined impacts of habitat degradation and recurrent cyclones on a community of small mammals in a rainforest landscape in Madagascar. We used capture-release and morphometry data of 609 individuals of shrew tenrecs, rodents, and nocturnal lemurs, and vegetation surveys from 360 plots in four sites with different degradation levels for four field seasons (2021–2023) separated by two cyclone events. Combined impacts of degradation and cyclones significantly affected small mammals’ diversity and capture abundance and only the body mass of the lesser tufted-tailed rat and brown mouse lemur. Diversity, capture abundance and body mass decreased immediately after the cyclones, and bounced back 4–5 months later, except in the forest fragment. We also examined the independent effects of habitat degradation using vegetation structure as it had more impacts than cyclones on small mammals. Plant diversity, canopy cover percentage, mean diameter at breast height, and estimated height significantly impacted small mammals’ diversity, capture abundance, and body-mass with species-specific variations. Our results suggest that recurrent cyclones may act as an intermediate disturbance factor, while habitat degradation might have permanent impacts on small mammals, emphasizing the importance of long-term monitoring of wild populations to understand their spatiotemporal dynamics and their effective conservation.

## Introduction

Habitat transformation and climate change are among the main causes of biodiversity loss worldwide. Both restrict the number of species and individuals persisting in an area and modify biotic interactions, altering the stability and function of many ecosystems^1,2^. Recurrent or extreme climatic events, such as cyclones and droughts, play important roles in shaping ecosystem dynamics in the tropics^1,3^. They not only change the composition and structure of plant communities via defoliation and uprooting, but also disturb the dynamics and services of ecosystems^1,3–5^, and consequently, the occurrence and survival of animal communities^3,4^. Also, prolonged exposure to light from the newly opened canopy gaps resulting from such climatic events can prevent some plant species from sprouting, which might restrict animal movement and dispersal^1,3^. Finaly, the thinning or absence of woody debris and leaf litter from cyclone runoffs might also lead to inadequate microhabitats for ground-dwelling animals^1,5^.

Recurrent or extreme climatic events may exacerbate the negative impacts of habitat transformation on communities. For instance, fragmented or severely degraded forests can take a longer time to recover after cyclones and ecological successions are often halted or disturbed^1,6^. However, we have little understanding of the extent to which the combination of habitat degradation and recurrent climatic events affects the animal communities (but see^5^). Looking at the impacts of habitat degradation and climatic events together can thus provide a holistic view of the vulnerability, resilience, and adaptability of biological communities and ecosystems. Uncovering such impacts can also help practitioners build more sustainable and resilient conservation and mitigation measures, especially as we expect an increase in the frequency and intensity of climatic events in multiple regions worldwide^2^.

Here, we seek to uncover the combined effects of habitat transformation and recurrent climatic events on animal communities in Madagascar. We examined the combined impacts of habitat degradation and tropical cyclones on the diversity, population size, and body mass of small-bodied mammals. We hypothesized that habitat degradation and cyclones will have synergistic negative impacts on small mammal communities, due to resource limitation and alterations to forest structure and composition^1,5^. As a result, we expect significant declines in small mammal diversity, population size, and body mass, with greater magnitude in the most disturbed forest habitats. We then examined the independent impacts of habitat degradation, using plant diversity, size (diameter and height), and canopy cover percentage, on the small mammal community structure.

The island of Madagascar provides a useful test case for such research objectives for the following reasons. First, most of its habitats and landscapes are experiencing ongoing transformation, negatively impacting the occurrence and survival of some animal species^7^. Second, it experiences recurrent cyclones, which are known to influence the forest dynamics and the structure of animal communities^8,9^. Finally, its animal communities are known to have different responses to disturbances (e.g.,^10–12^). We conducted this study in four sites within and around Ranomafana National Park (RNP; Fig. S1), southeastern Madagascar from July 2021 to August 2023. RNP rainforest landscape constitutes an ideal location to address our research goal because its forest habitats have different levels of degradation depending on the legal protection in place and resource use, and it experiences frequent cyclones every year^8^. Two category-4 cyclones (Batsirai and Emnati with an average wind gust > 225 km/h^13^) made landfall in southeastern Madagascar in February 2022 with only a 17 days interval, and another in February 2023 (Freddy, category 3 with wind gust > 150 km/h); https://www.meteoblue.com/historyplus/).

We focused on the community of small mammals in this study because they are often considered resilient to disturbances (but see^14^) and thus, are usually the last extirpated from a habitat^5,15^, offering an opportunity to observe their responses to ecological changes. The community of small mammals in RNP includes mainly nocturnal lemurs (Groves’ dwarf lemurs, *Cheirogaleus grovesi* and brown mouse lemurs, *Microcebus rufus*)^16^, native rodents (*Eliurus minor*, *E. tanala*, *E. webbi*, *Nesomys audeberti*, and *N. rufus*), hedgehog tenrecs (*Setifer setosus* and *Tenrec ecaudatus*), 14 species of shrew tenrecs (genus: *Microgale*), and invasive rodents (*Rattus norvegivus* and *R. rattus*)^17^. Other native small mammal species are present in RNP but are not commonly captured in live traps^17^, thus, not listed here. Only the lemurs and some species of shrew tenrecs are on the list of threatened species^18^. All rodents and hedgehog tenrecs are predominantly ground dwelling, while the lemurs and shrew tenrecs are mostly arboreal^16,17^.

## Results

We captured 14 species of small mammals over the course of our study (Table 1). In total, we captured-released 1021 individuals of shrew tenrecs, native and invasive rodents, and nocturnal lemurs over 4320 night-traps (3 cyclone status × 4 sites × 2 transects × 4 nights × 45 traps). We excluded 8 small mammal species that were observed only once, 469 recaptured individuals and 37 body mass measurement outliers, giving a total of 515 individuals for the subsequent analyses.

**Table 1:** Small mammal species surveyed within and around Ranomafana National Park in 2021–2023 with their respective capture number.

Family	Species	IUCN status	Total capture
Cheirogaleidae	Cheirogaleus grovesi*	VU	1
	*Microcebus rufus*	VU	328 (264)
Nesomyidae	Brachytarsomys albiicauda*	LC	1
	*Eliurus minor*	LC	207 (66)
	*Eliurus tanala*	LC	107 (51)
	*Eliurus webbi*	LC	135 (37)
	Nesomys audeberti*	LC	7
	Nesomys rufus*	LC	10
Muridae	Rattus norvegicus*	LC	2
	*Rattus rattus*	LC	207 (85)
Tenrecidae	Microgale fotsifotsy*	LC	2
	Microgale longicaudata*	LC	1
	*Microgale principula*	LC	12 (12)
	Microgale talazaci*	LC	1
Total			1021 (515)

Conservation status according to the International Union for Conservation of Nature (2024): LC, Least Concern; VU, Vulnerable. “*” indicates that the mall mammal species were excluded from capture abundance and body mass analyses because of low observations. Values in brackets indicate the number of individuals included in analyses after exclusion of recapture and outliers.

### Combined impacts of habitat degradation and cyclones

We fitted Linear Mixed-effect Models in R 4.4.1^19^ to assess the combined impacts of habitat degradation and cyclones on the small mammal community: diversity (one model), capture abundance and body mass (six models each). Diversity refers to Shannon’s diversity index of the small mammals; and capture abundance, which is a proxy for population size^20^, refers to the number of individuals captured per small mammal species for every 45 traps deployed. Body mass is from the systematic weighing of each captured small mammal^17,21^. We used the value per transect for each of these community structure metrics (3 cyclone status × 4 habitat types × 2 transects = 24 values). We set habitat types and cyclone status as fixed effects and transect and season as random effects. As reproductive state can also affect body mass^15,22^, we added reproduction signs as a random effect when fitting the body mass of small mammals. We used the forest habitat types as a proxy for increasing habitat degradation levels: primary forest, secondary forest, forest fragment, and crop field^12^; see Methods for further details). We used cyclone status as a proxy for recurrent climatic events: “before”, “immediate”, and “4–5 months”, which respectively refer to before the cyclones (August–September), immediately after the cyclones (February–March), and 4–5 months after the cyclones (June–July; see Methods for further details).

#### Diversity

**Fig. 1:**
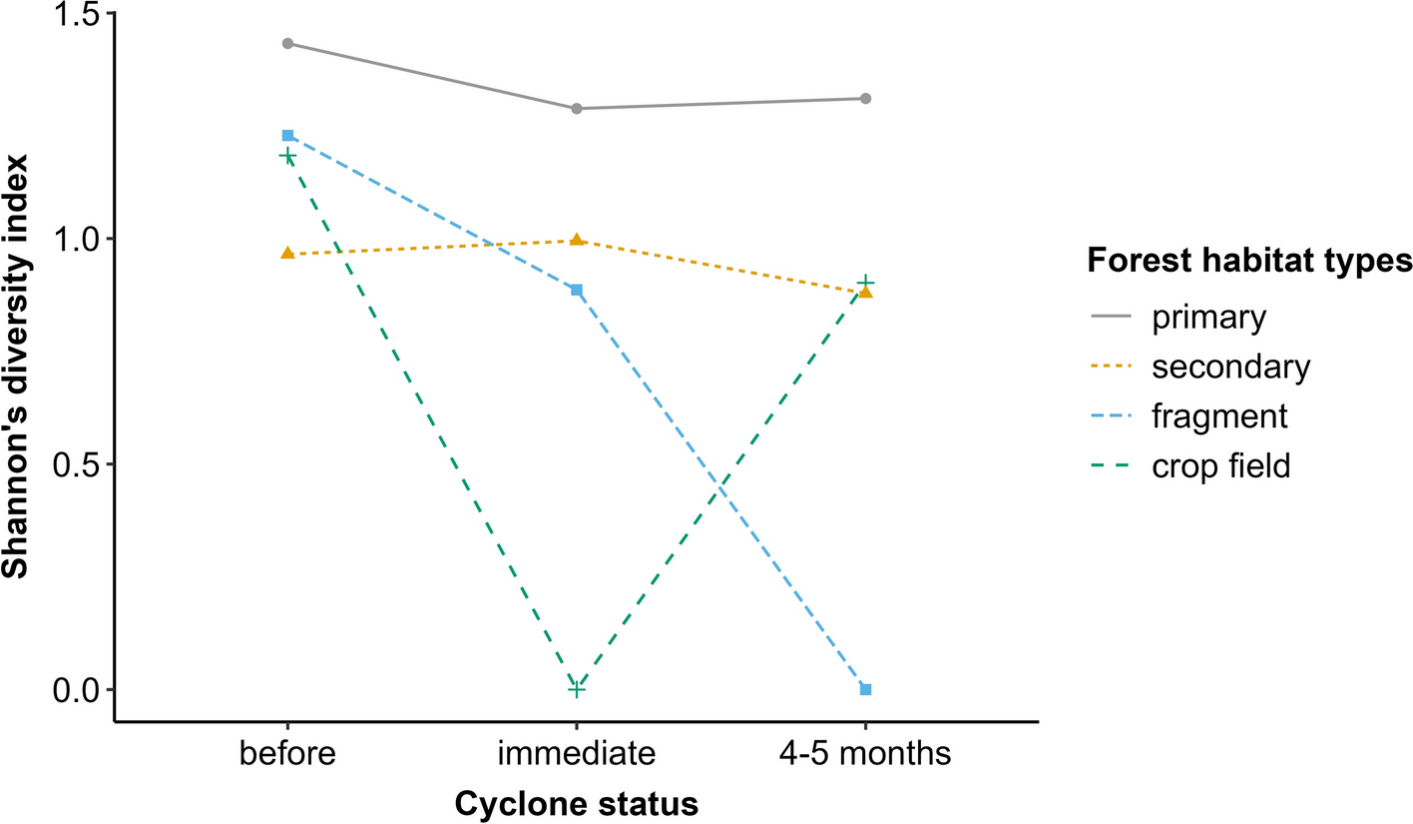
Changes in the diversity of small mammals within and around Ranomafana National Park in 2021–2023.

The diversity of small mammals decreased immediately after the cyclones, then recovered 4–5 months after the cyclones, except in the secondary forest and forest fragment (Fig. 1). Habitat degradation and cyclones significantly decreased the diversity of small mammals immediately after the cyclones in the primary forest but increased it in the secondary forest (Fig. 1, Table S1).

#### Capture abundance

**Fig. 2:**
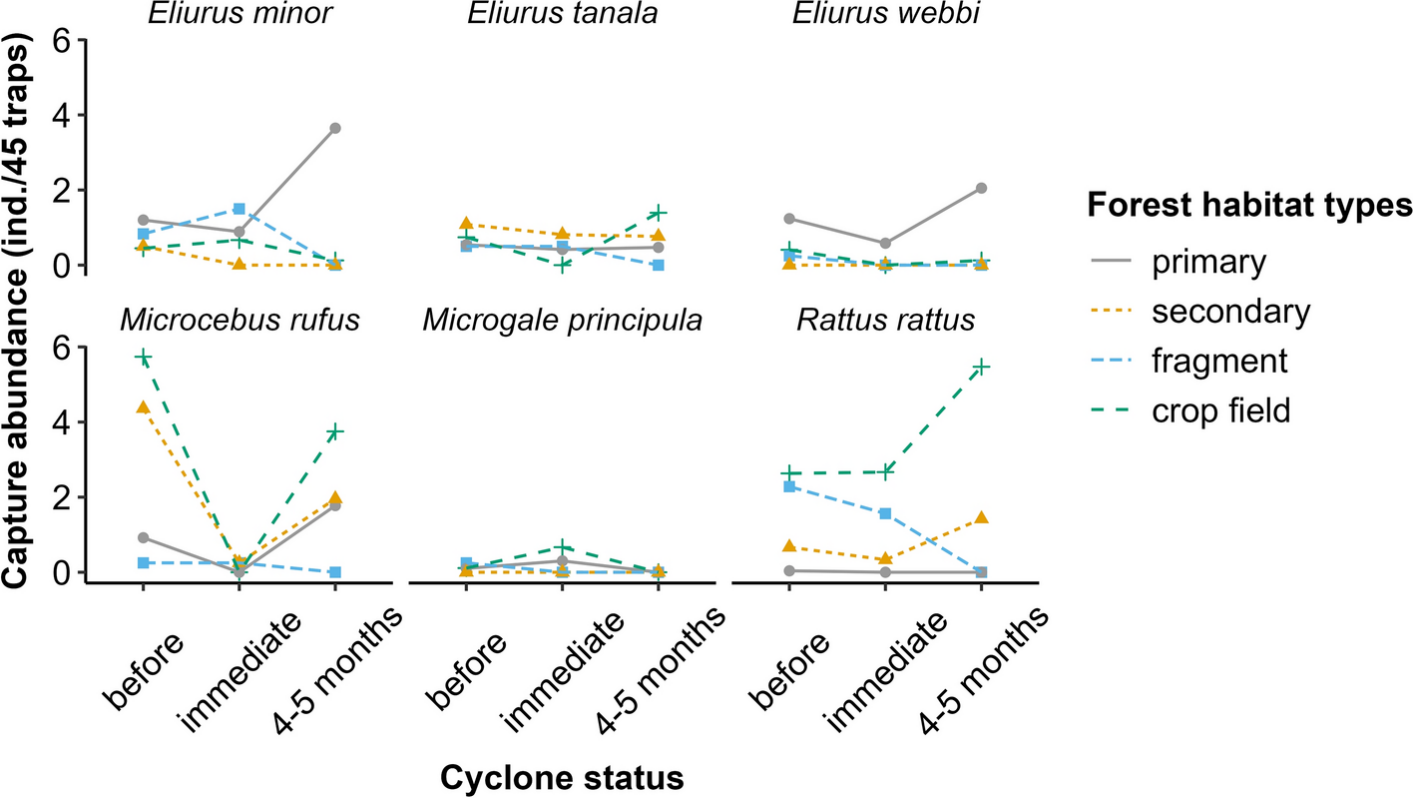
Changes in the capture abundance of small mammals within and around Ranomafana National Park in 2021–2023. Absence of a species is indicated by zero values.

The capture abundance of most small mammal species was significantly higher before the cyclones in all forest habitat types (Fig. 2; Table S1). It then significantly decreased in primary forest for the lesser and tanala tufted-tailed rats (*Eliurus minor* and *E. tanala*) and in the secondary forest and the crop field for the black rat (*Rattus rattus*). The capture abundance of all small mammal species bounced back to their initial state or even higher 4–5 months after the cyclones, though not significantly. Data from the forest fragment 4–5 months after the cyclones are missing (represented by capture abundance = 0 in Fig. 2) since the forest patch has been wiped out by the cyclones in 2022. It is also worth noting that no individuals of the greater long-tailed tenrec shrew (*Microgale principula*) were captured after the cyclones in any forest habitat types despite a significant increase of their capture abundance in the primary forest and crop field. *Rattus rattus* was present in the primary forest before the cyclones with only one individual, then remained absent. However, it started to be present in the crop field immediately after the cyclones.

#### Body mass

**Fig. 3:**
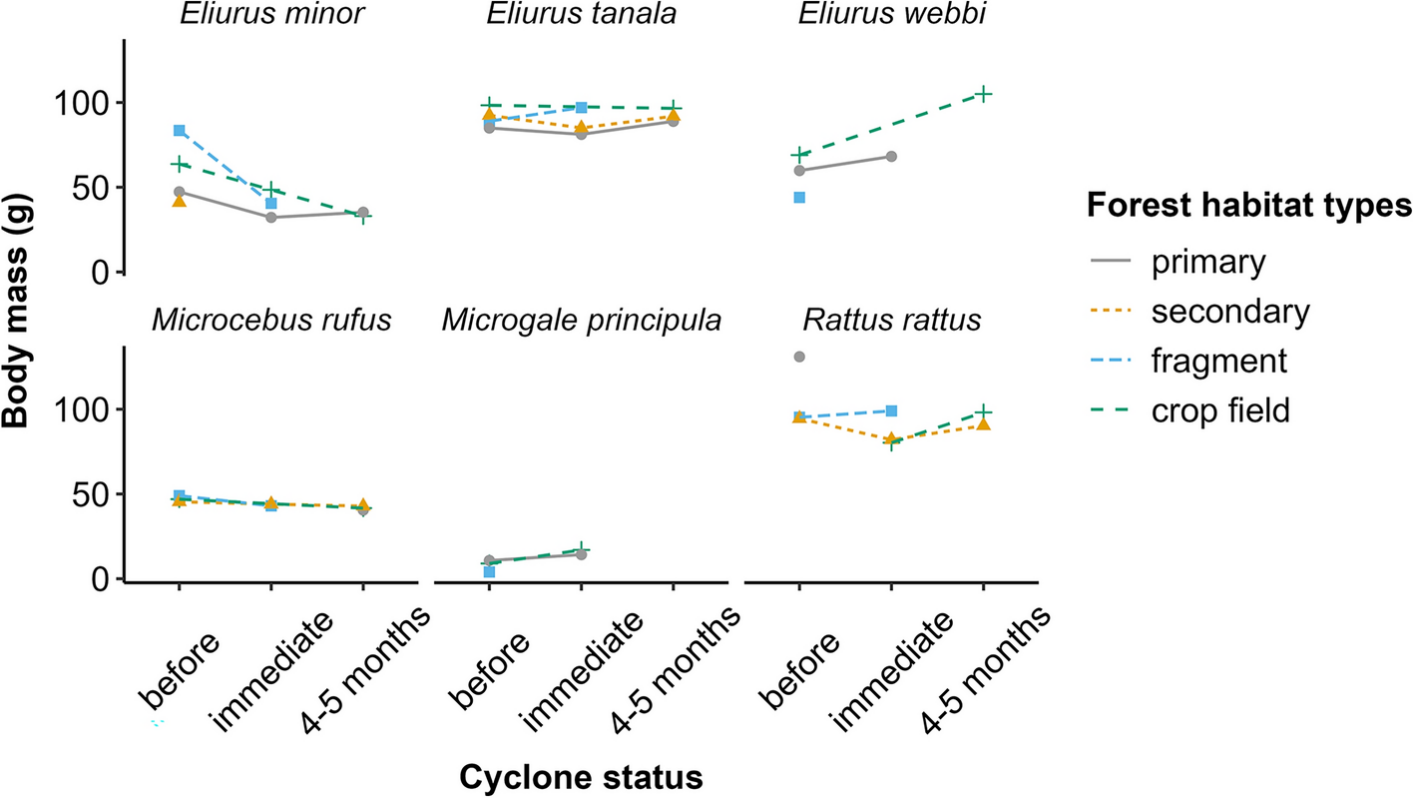
Changes in the body mass of small mammals within and around Ranomafana National Park in 2021–2023. Absence of a species is indicated by missing values.

The body mass of all small mammal species, except Webb’s tufted-tailed rat (*Eliurus webbi*) and *Microgale principula*, significantly decreased immediately after the cyclones (Fig. 3; Table S1). Body mass only bounced back 4–5 months after the cyclones in primary and secondary forests for two tufted-tailed rats (*Eliurus minor* and *E. tanala*), and significantly in the secondary forest and crop field for *R. rattus*. It is worth noting that conditional R^2^ (R^2^c) were higher than marginal R^2^ (R^2^m) when fitting LMM models with body mass, indicating that seasons and reproductive state play a major role in the fluctuation of body mass of small mammals, in addition to only habitat degradation and cyclones.

In summary, we found that capture abundance of small mammals was more sensitive to the combined effects of habitat degradation and recurrent cyclones than body mass. Additionally, both capture abundance and body mass were more affected by the forest habitat types than the cyclones (Table S1).

### Independent impacts of habitat degradation

We performed stepwise Linear Mixed-effect Models with *both* model selection of fixed and random effects using the function *step* in the R-package “lmerTest” to assess the impacts of habitat degradation on the structure of small mammals based on vegetation structure^23^. We used plant diversity, canopy cover percentage, mean diameter at breast height (DBH), and estimated height as proxies for habitat degradation level^10^, calculated from the survey of 360 5 × 5 botanical plots^12^ around each trap deployed across the four sites (45 traps × 2 transects × 4 sites). Plant diversity refers to the Shannon’s diversity index of surveyed plants per plot. We calculated canopy cover percentage from the estimated leaf crown diameter^24^, mean DBH, and mean estimated height of per plot (see Methods for further details). We then averaged these values per transect such that we have the same number of observations as the small mammals’ diversity, capture abundance, and body mass (2 transects × 4 sites = 8 values). We fitted the same number of models as above. We set plant diversity, canopy cover, mean DBH, and mean estimated height as fixed effects, and transect and plot as random effects. We added reproduction signs as a random effect when fitting the body mass of small mammals. Significant predictors of the small mammal community metrics have a *p*-value < 0.05.

#### Diversity

Small mammals’ diversity significantly increased with plant diversity, mean DBH, and estimated height (Fig. S2). However, it decreased with high canopy cover percentages (Fig. S1).

#### Capture abundance

Habitat degradation affected each small mammal species in different ways (Figs. S3–S6 top panel). The capture abundance of most species, except that of *Eliurus minor* and *Microgale principula*, significantly increased with plant diversity. We observed similar patterns for canopy cover percentage in all species but *M. principula*; and for mean DBH and estimated height, except for the brown mouse lemur (*Microcebus rufus*), *Microgale principula*, and *R. rattus*.

#### Body mass

Different habitat proxies differently impacted the body mass of each small mammal species (Figs. S3–S6 bottom panel). Body mass significantly increased with plant diversity in all species but *Eliurus webbi* and *R. rattus*; and with canopy cover percentage in all species but *E. minor*. However, body mass significantly decreased with increasing mean DBH in all species but *Microgale principula*. We found similar patterns for mean estimated height in all species, except *M. principula* and *Rattus rattus*.

In summary, high plant diversity and canopy cover with small-diameter (DBH < 5 cm) and understory plants (height < 4.5 m) maintain a high diversity and population size of small mammals. Although we observed similar patterns for body mass, other external factors associated with transects and plots as well as the reproductive state of the individuals and seasonality seem to influence the body mass variability.

## Discussion

### Combined impacts of habitat degradation and recurrent cyclones

We found that the combination of habitat degradation and recurrent cyclones significantly decreased the diversity, population size, and body mass of small mammals in RNP immediately after the cyclones with more noticeable difference in the primary forest and crop field, partially validating our hypothesis. Diversity, population size, and body mass bounced back 4–5 months after the cyclones for most species across forest habitat types, except those in the forest fragment, indicating a certain level of resilience in the small mammals. When looking at the independent impacts of habitat degradation, we found that plant diversity and size as well as canopy cover mediated the diversity, population and body size of small mammals with different variations from one species to another. Such results indicate that each small mammal species has their own coping mechanisms in the face of habitat degradation, calling for more ecological information about each species and probably a species-centered conservation approach. Finally, we observed that diversity and capture abundance were more vulnerable to the combined disturbances and to habitat degradation than body mass, which highlights that body mass fluctuations depend on both internal and external conditions to the species and individuals^15,22^.

A decrease in animal population size and body mass is a common impact of habitat transformation and recurrent climatic events^5,25^, suggesting that these two disturbances may amplify each other’s negative impacts. Here, we observed this immediately after the cyclones, when the resources temporarily become restricted^9,26^. However, populations and individuals in the crop field unexpectedly recovered faster and at a greater magnitude than those in the other forest habitats. We can associate such resilience with the abundance of food resources from the different types of crops available year-round (e.g., banana, coffee, cassava, lychee, etc.), on which the small mammals can rely for their subsistence. Alternatively, predators such as carnivores and birds might be rare in disturbed forests, relieving some ecological pressure on the small mammal community^5,15,26^. In primary and secondary forests, spatial and food resources may gradually increase after the cyclones, offering small mammals a slower recovery. Our findings thus suggest that small mammals can maintain their structure and body conditions despite such recurrent climatic events. Therefore, cyclones could be considered as an intermediate disturbance factor structuring the population dynamics and body conditions of animals in tropical ecosystems, i.e., recurrent stress from cyclones favour fast-growing populations and individuals^1,26–29^.

### Resilience of small mammals

The ability of small mammals to recover after disturbances in this study suggests that they may have intrinsic characteristics that allow them to cope well with disturbances. For instance, their small body size could allow them to find shelter in any opening in the forests, thus making them less vulnerable to the loss and damage of their (micro)habitats^15,17,30^. Additionally, their small body size could facilitate their escape from threats and allow easier dispersal to a safer area^1,3,5,30^. Also, given that small mammals are often omnivorous^16,17^, they could rely on any food sources available that would allow them to rapidly recover from the disturbances^30,31^. Moreover, small mammals are often ground dwelling or use the lower strata of the forests, which are usually less affected by cyclones and habitat degradation^27,32^, and thus, could continue providing them with their required niche^3,31,32^. Finally, like their larger-bodied counterparts, small mammals may synchronize their seasonal reproduction period with the recurrent disturbances such that their population dynamics would not be substantially interrupted (e.g.^3,8^). We therefore argue that most small mammals in this system are ecologically flexible enough to overcome the challenges of short-term recurrent climatic events and habitat transformation. Nevertheless, this study also hints that some small mammal species recover faster when habitat structures are maintained (e.g., in primary forest) or food resources are available (e.g., in crop field), stressing the importance of protecting natural habitats and making human-modified habitats more friendly to animals^33,34^.

### Implications for conservation

This study shows the opportunistic traits of invasive animals. Here, we found that *Rattus rattus,* an introduced rodent species that has significantly spread across Madagascar^17,20,35^, grew rapidly after the cyclones in all habitats but the primary forest, where it disappeared. However, closed canopy with large-diameter and tall plants seem to inhibit their occurrence and growth^20,35^. Therefore, maintaining moderately and severely degraded forests may be necessary to prevent *R. rattus* from moving further into the forest, or at least using their edges, and thus negatively interacting with the native species.

Our results showed that the impacts of habitat degradation and cyclones affected each species of small mammals differently. More interestingly, animal presence was associated with better habitat quality, suggesting that they might require specific niches to maintain their population sizes^17,25,32^. It is also worth noting that some species of nocturnal lemurs and tenrecs are heterothermic (i.e., able to modulate their body temperatures and activities according to the ambient temperatures via daily torpor or hibernation), and thus might not be active during the cold season^36^. Such ecophysiological strategy may have reduced their capture abundance during this study, warranting further investigation. As for the nocturnal primates, the populations and individuals in secondary forests and crop fields were relatively resilient to changes, probably because of either the fruiting peak of the invasive strawberry guava (*Psidium cattleianum*) in the secondary forests of RNP^37^ or the continuous availability of crop resources might contribute to the maintenance of their populations and individuals after the cyclones. Therefore, there is a need for more species-centred conservation approach to protect animal populations in the wild^33,34^. Additionally, we encourage including small mammals in long-term monitoring of biodiversity given their population and individual spatiotemporal fluctuations. Such data could capture intra-and inter-annual dynamics of their populations and individuals, disentangle eventual driving forces of their structure and responses, and help predict and mitigate ecological changes’ consequences.

## Methods

### Site description

Ranomafana National Park (21° 16′ S and 47° 20′ E) and its surroundings display a landscape of montane and mid-elevation rainforest with various degrees of anthropogenic modification^12,38^. The primary forest in Valohoaka is part of the largest forest block of RNP and is dominated by a montane rainforest^12,38^. It is also legally protected without resources extraction. Vohiparara is also legally protected but at the edge of the forest block and is considered a secondary forest. Its original vegetation has entirely been replaced by a new assemblage of plants with an increasing population of invasive plant species^12^. Bevoahazo is a forest patch adjacent to the RNP legal limit and is under controlled use by the local community (i.e., undergoes selective woody and non-woody product extraction)^12^. Finally, Ankazomasina is a privately owned plantation land, where the buffer zones of RNP (forest edges) overlap with multiple stands of crop species (e.g., banana, coffee, cassava, and lychee; VR unpublished data).

During our study period, daily temperatures and precipitation in RNP ranged between 8–29 °C and 24–696 mm, respectively (https://www.meteoblue.com/historyplus/). RNP undergoes two main seasons^39^ : a dry season from May to September with a respective daily temperature and precipitation of 11.46–20.59 °C and 89.09–221.60 mm; and a rainy season from October to April with a respective daily temperature and precipitation of 14.08–25.91 °C and 372–1321 mm (https://www.meteoblue.com/historyplus/). Cyclone status in Madagascar generally occurs between November and March and can influence the precipitation of the early dry season^9,11^. Therefore, we classified our cyclonic period in this study as follows: “before”, “immediate”, and 4–5 months after the cyclones. “Before” (August–September) is the end of the dry season and the precipitation is not influenced by the cyclonic season; “Immediate” (February–March) starts 1–2 weeks after the cyclones; and “4–5 months” (June–July) is the start of the dry season, during which the cyclonic season can still influence the precipitation.

### Capture abundance and body mass of small mammals

Following the protocol in Ramananjato et al.^21^, we set up 45 banana-baited live traps (35 LFA 7.63 × 8.89 × 22.86 cm, and 10 LFA 10.16 × 11.42 × 38.10 cm Aluminium traps, Sherman traps, Inc.) every 25 m along a non-linear transect for four consecutive nights at 0–2.5 m above the ground. We alternated capture-release in two transects per site. We identified the small mammal species using local field guides developed by Soarimalala and Goodman^17^ and Mittermeier et al.^16,40^. We marked each captured individual with a unique color code using special animal markers (Markal Paintstick). Finally, we systematically measured the body mass and recorded any signs of reproduction (lactation, gestation, trace of semen, etc.) of each captured individual during the study.

### Habitat degradation proxies

Following the protocol in Ramananjato and Razafindratsima^12^, we surveyed 360 botanical plots of 5 × 5 m that were set up around each live trap deployed (45 plots × 4 sites × 2 transects). In each plot, we identified each plant according to its vernacular name, which we later matched with the local plant database, estimated its height and crown diameter, and measured its Diameter at breast height (DBH). We calculated the canopy cover percentage from the estimated leaf crown diameter of each plant surveyed, using the formula from Gray et al.^24^: CC = 100e^−0.01SC^, with CC as the canopy cover percentage and SC the canopy cover percentage based on the sum of visually estimated crown diameter.

## Supplementary Information

Supplementary Information.

## Data Availability

Data generated and analysed in this study have been deposited in figshare and will be made publicly available upon manuscript acceptance. 10.6084/m9.figshare.27702870. The point of contact is Veronarindra Ramananjato: veronarindra@berkeley.edu.
