# Supplementary material for: Combined impacts of habitat degradation and cyclones on a community of small mammals
Source: Sci Rep. 2025 May 14;15:16760. doi: 10.1038/s41598-025-00740-w (PMC12078653; doi:10.1038/s41598-025-00740-w)
Supplement: Supplementary file 1 — Supplementary Information. [file 41598_2025_740_MOESM1_ESM.pdf]

# SUPPLEMENTARY MATERIALS

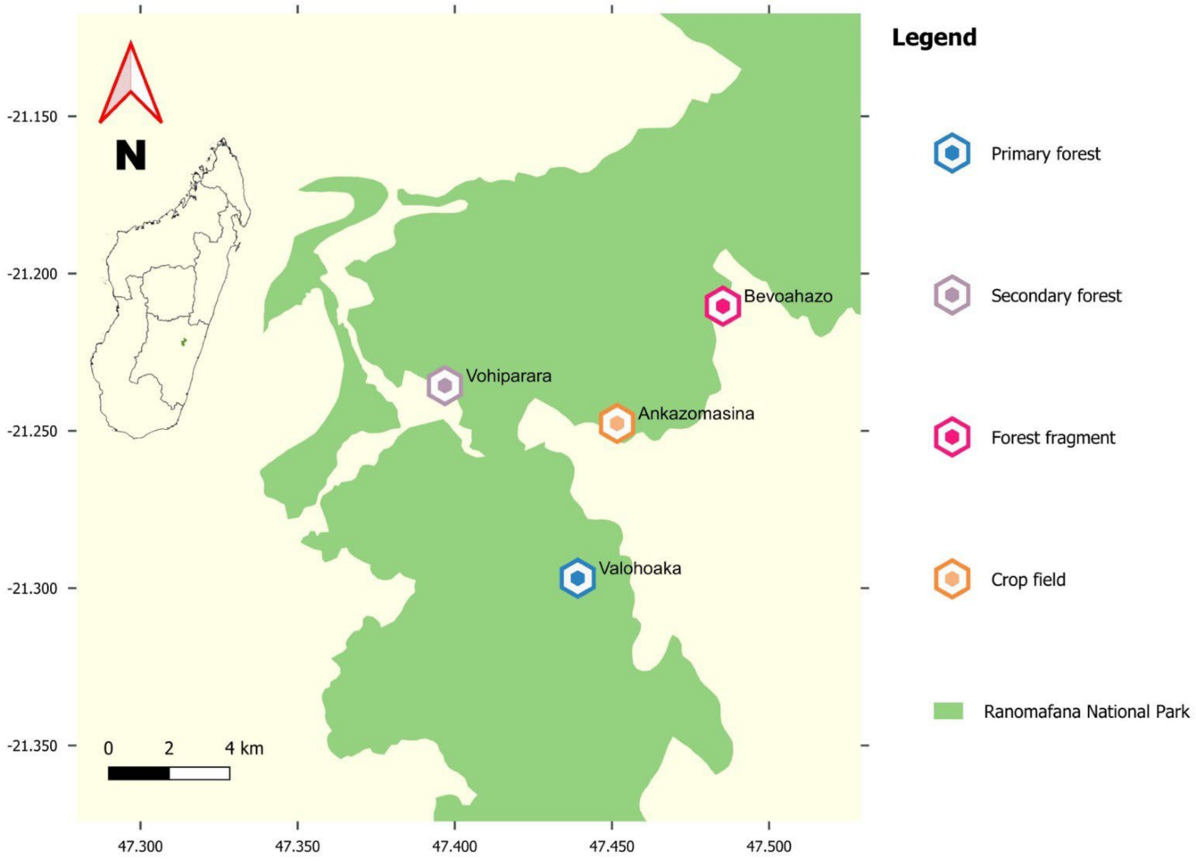


**Figure S1**. Location of the four sampling sites within and around Ranomafana National Park. Source: Foiben-Taotsarintanin'i Madagasikara (FTM) and Réseau de la Biodiversité Malgache (www.rebioma.net).

**Table S1**. Summary table of the Linear Mixed-effect Models of the combined effects of habitat degradation and recurrent cyclones on the small mammal community within and around Ranomafana National Park in 2021-2023. Forest habitat types: PR = primary forest, SC = secondary forest, FG = forest fragment, and CR = crop field. LMM statistics: β estimates of the models; t = fitted models; p = probability of deviation; $R^2M$ = marginal $R^2$, which is the proportion of variances explained by the fixed factors alone; and $R^2C$ = conditional $R^2$, which is the proportion of variances explained by both the fixed and random factors.

| | Before cyclones | | | | Immediately after cyclones | | | | 4-5months after cyclones | | | | LMM $R^2$s |
|---|---|---|---|---|---|---|---|---|---|---|---|---|---|
| | PR | SC | FG | CR | PR | SC | FG | CR | PR | SC | FG | CR | |
| **Diversity** | ß = -0.160 | ß = -0.195 | ß = -0.077 | ß = 0.090 | ß = 0.114 | ß = 0.253 | ß = -0.137 | ß = -0.137 | ß = 0.282 | ß = 0.282 | ß = 0.282 | ß = 0.282 | |
| | t = -4.757 | t = -5.800 | t = -2.029 | t = 8.649 | t = 3.013 | t = 6.667 | t = -4.620 | t = -4.620 | t = 11.849 | t = 11.849 | t = 11.849 | t = 11.849 | $R^2m$ = 0.481; $R^2c$ = 0.728 |
| | p < 0.001 | p < 0.001 | p < 0.050 | p = 0.066 | p < 0.01 | p < 0.001 | p < 0.001 | p < 0.001 | p = 0.323 | p = 0.323 | p = 0.323 | p = 0.323 | |
| **Capture abundance** | | | | | | | | | | | | | |
| ***Eliurus minor*** | ß = -3.990 | ß = 0.597 | ß = -1.092 | ß = -0.639 | ß = -5.025 | ß = 0.597 | ß = 0.833 | ß = -0.639 | ß = 3.989 | ß = 0.597 | ß = -0.639 | ß = -0.639 | |
| | t = -6.823 | t = 1.380 | t = -2.052 | t = - 0.593 | t = -7.437 | t = 1.380 | t = 1.907 | t = - 0.593 | t = 7.412 | t = 1.380 | t = - 0.593 | t = - 0.593 | $R^2m$ = 0.447; $R^2c$ = 0.919 |
| | p < 0.001 | p = 0.169 | p < 0.050 | p = 0.613 | p < 0.001 | p = 0.169 | p = 0.058 | p = 0.613 | p < 0.001 | p = 0.169 | p = 0.613 | p = 0.613 | |
| ***Eliurus tanala*** | ß = 0.700 | ß = 0.963 | ß = 0.749 | ß = -0.609 | ß = -0.187 | ß = 0.963 | ß = -0.982 | ß = 0.078 | ß = 1.341 | ß = -0.639 | ß = -0.982 | ß = 1.341 | |
| | t = 6.818 | t = 7.911 | t = 3.364 | t = -7.703 | t = -1.188 | t = 7.911 | t = -5.135 | t = 0.683 | t = 15.094 | t = -8.114 | t = -5.135 | t = 15.094 | $R^2m$ = 0.628; $R^2c$ = 0.695 |
| | p < 0.001 | p < 0.001 | p < 0.01 | p < 0.001 | p = 0.238 | p < 0.001 | p < 0.001 | p = 0.496 | p < 0.01 | p < 0.001 | p < 0.001 | p < 0.01 | |
| ***Eliurus webbi*** | ß = -1.128 | N/A | ß = 0.422 | ß = 0.422 | ß = -1.479 | N/A | ß = -1.479 | ß = -1.479 | ß = 2.010 | N/A | ß = 0.071 | ß = -0.059 | |
| | t = -2.021 | N/A | t = -0.767 | t = -0.767 | t = -4.135 | N/A | t = -4.135 | t = -4.135 | t = 3.848 | N/A | t = 0.128 | t = -0.999 | $R^2m$ =0.445; $R^2c$ = 0.617 |
| | p < 0.050 | N/A | p = 0.444 | p = 0.444 | p < 0.001 | N/A | p < 0.001 | p < 0.001 | p < 0.001 | N/A | p = 0.898 | p = 0.921 | |
| ***Microcebus rufus*** | ß = -3.045 | ß = 0.073 | ß = -4.175 | ß = 2.129 | ß = -1.914 | ß = -1.598 | ß = -1.020 | ß = -2.008 | ß = 3.587 | ß = 3.587 | N/A | ß = 3.587 | |
| | t = -15.030 | t = 0.307 | t = -3.246 | t = 16.609 | t = -13.368 | t = -10.229 | t = -0.965 | t = -2.631 | t = 10.765 | t = 10.765 | N/A | t = 10.765 | $R^2m$ = 0.832; $R^2c$ = 0.875 |
| | p < 0.001 | p = 0.759 | p < 0.001 | p < 0.001 | p < 0.001 | p < 0.001 | p = 0.335 | p = 0.185 | p =0.558 | p =0.558 | N/A | p = 0.558 | |
| ***Microgale principula*** | ß = 0.049 | ß = 0.072 | ß = 0.139 | ß = 0.072 | ß = -0.394 | ß = 0.555 | ß = 0.555 | ß = 0.555 | N/A | N/A | N/A | N/A | |
| | t = 0.470 | t = 0.271 | t = 1.140 | t = 0.271 | t = -3.155 | t = 1.533 | t = 1.533 | t = 1.533 | N/A | N/A | N/A | N/A | $R^2m$ = 0.327; $R^2c$ = 0.931 |
| | p = 0.653 | p = 0.794 | p = 0.298 | p = 0.794 | p < 0.050 | p = 0.176 | p = 0.176 | p = 0.176 | N/A | N/A | N/A | N/A | |
| ***Rattus rattus*** | ß = -2.914 | ß = 2.159 | ß = -2.914 | ß = -2.914 | ß = -2.520 | ß = 1.718 | ß = -3.116 | ß = -3.116 | ß = 5.627 | ß = 5.627 | ß = -1.104 | ß = 5.627 | |
| | t = -28.410 | t = 5.489 | t = -28.410 | t = -28.410 | t = -4.764 | t = 3.048 | t = -12.427 | t = -12.427 | t = 28.156 | t = 28.156 | t = -4.231 | t = 28.156 | $R^2m$ = 0.904; $R^2c$ = 0.922 |
| | p < 0.001 | p < 0.001 | p < 0.001 | p < 0.001 | p < 0.001 | p < 0.01 | p < 0.001 | p < 0.001 | p < 0.050 | p < 0.050 | p < 0.001 | p < 0.050 | |
| **Body mass** | | | | | | | | | | | | | |
| ***Eliurus minor*** | ß = 32.126 | ß = 2.292 | ß = 2.292 | ß = 2.292 | ß =-30.774 | ß =-30.774 | ß =-30.774 | ß =-30.774 | ß = 65.917 | ß = -7.861 | ß = -7.861 | ß = -7.861 | |
| | t = 2.417 | t = 0.175 | t = 0.175 | t = 0.175 | t =-1.999 | t =-1.999 | t =-1.999 | t =-1.999 | t = 2.695 | t =-0.513 | t =-0.513 | t =-0.513 | $R^2m$ = 0.286; $R^2c$ = 0.870 |
| | p < 0.050 | p = 0862 | p = 0862 | p = 0862 | p = 0.053 | p = 0.053 | p = 0.053 | p = 0.053 | p = 0.135 | p = 0.610 | p = 0.610 | p = 0.610 | |
| ***Eliurus tanala*** | ß = 4.790 | ß = 16.571 | ß = 0.570 | ß = 0.570 | ß = 0.557 | ß = -18.500 | ß = 7.273 | ß = 7.273 | ß = 100.415 | ß = -18.50( | N/A | ß =-14.574 | |
| | t = 0.064 | t = 1.152 | t = 0.079 | t = 0.079 | t = 0.291 | t = -1.429 | t = 0.501 | t = 0.501 | t =7.705 | t = -1.429 | N/A | t = -1.992 | $R^2m$ = 0.111; $R^2c$ = 0.677 |
| | p = 0.949 | p = 0.256 | p = 0.937 | p = 0.937 | p 0.773 | p = 0.161 | p = 0.622 | p = 0.622 | p < 0.050 | p = 0.161 | N/A | p = 0.553 | |
| ***Eliurus webbi*** | ß = 31.816 | N/A | ß =-31.579 | ß =-31.579 | ß = -5.786 | N/A | ß = -5.786 | ß = -5.786 | ß = 116.121 | N/A | ß = 116.12 | ß = 116.121 | |
| | t = 1.656 | N/A | t = -1.714 | t = -1.714 | t = -0.329 | N/A | t = -0.329 | t = -0.329 | t = 5.854 | N/A | t = 5.854 | t = 5.854 | $R^2m$ = 0.207; $R^2c$ = 0.553 |
| | p = 0.109 | N/A | p = 0.097 | p = 0.097 | p = 0.745 | N/A | p = 0.745 | p = 0.745 | p < 0.001 | N/A | p < 0.001 | p < 0.001 | |
| ***Microcebus rufus*** | ß = -6.649 | ß = -4.551 | ß = 6.470 | ß = 6.470 | ß = 0.393 | ß = 0.393 | ß = 0.393 | ß = 0.393 | ß = -1.443 | ß = -2.497 | N/A | ß = 40.141 | |
| | t = -2.225 | t = -1.392 | t = 3.312 | t = 3.312 | t = 0.041 | t = 0.041 | t = 0.041 | t = 0.041 | t = -0.590 | t =-1.061 | N/A | t = 18.711 | $R^2m$ = 0.066; $R^2c$ = 0.090 |
| | p < 0.050 | p = 0.165 | p < 0.01 | p < 0.01 | p = 0.967 | p = 0.967 | p = 0.967 | p = 0.967 | p = 0 .557 | p = 0.290 | N/A | p < 0.001 | |
| ***Microgale principula*** | ß = 9.000 | ß = 9.000 | ß = 9.000 | ß = 9.000 | ß = -8.000 | ß = -8.000 | ß = -5.000 | ß = -8.000 | N/A | N/A | N/A | N/A | |
| | t = 1.165 | t = 1.165 | t = 1.165 | t = 1.165 | t = -0.691 | t = -0.691 | t = -0.514 | t = -0.691 | N/A | N/A | N/A | N/A | $R^2m$ = 0.229; $R^2c$ = 0.388 |
| | p = 0.999 | p = 0.999 | p = 0.999 | p = 0.999 | p = 0.999 | p = 0.999 | p = 0.643 | p = 0.999 | N/A | N/A | N/A | N/A | |
| ***Rattus rattus*** | ß = 11.452 | ß = -7.552 | ß = 11.452 | ß = 11.452 | N/A | ß = 17.818 | ß = 17.818 | ß = 17.818 | N/A | ß = -3.189 | N/A | ß = 94.070 | |
| | t = 1.435 | t = -0.399 | t = 1.435 | t = 1.435 | N/A | t = 0.526 | t = 0.526 | t = 0.526 | N/A | t = -0.459 | N/A | t = 3.559 | $R^2m$ = 0.035; $R^2c$ = 0.724 |
| | p = 0.155 | p = 0.487 | p = 0.155 | p = 0.155 | N/A | p = 0.601 | p = 0.601 | p = 0.601 | N/A | p = 0.647 | N/A | p = 0.095 | |

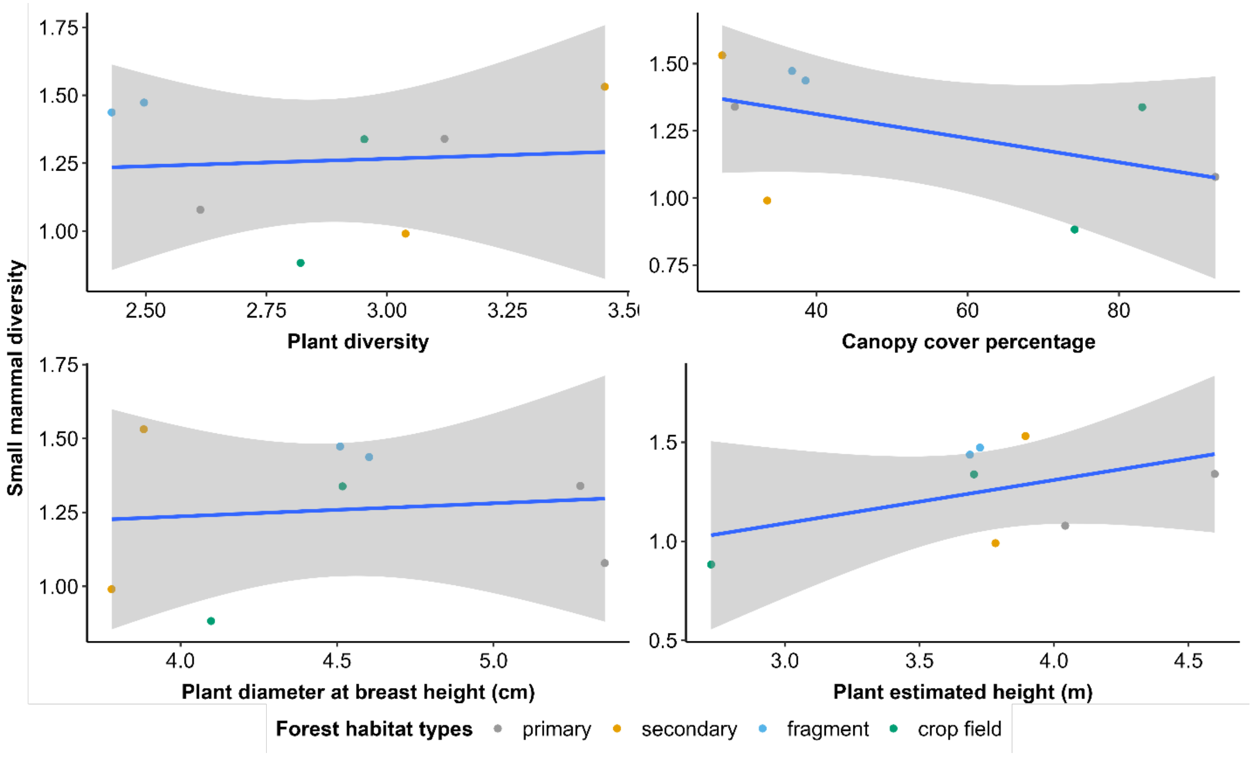


| Predictors | $\chi^2$ | F | p |
|---|---|---|---|
| **Plant diversity** | 3.156 | 174.787 | < 0.001 |
| **Canopy cover percentage** | 6.998 | 387.575 | < 0.001 |
| **Mean DBH** | 0.588 | 32.581 | < 0.001 |
| **Mean estimated height** | 0.957 | 53.032 | < 0.001 |

**Figure S2.** Changes in the diversity of small mammals within and around Ranomafana National Park in response to habitat degradation proxies with stepwise LMM coefficients: $\chi^2$ = sum of squares, F = model deviation from null model, and p = probability of deviation.

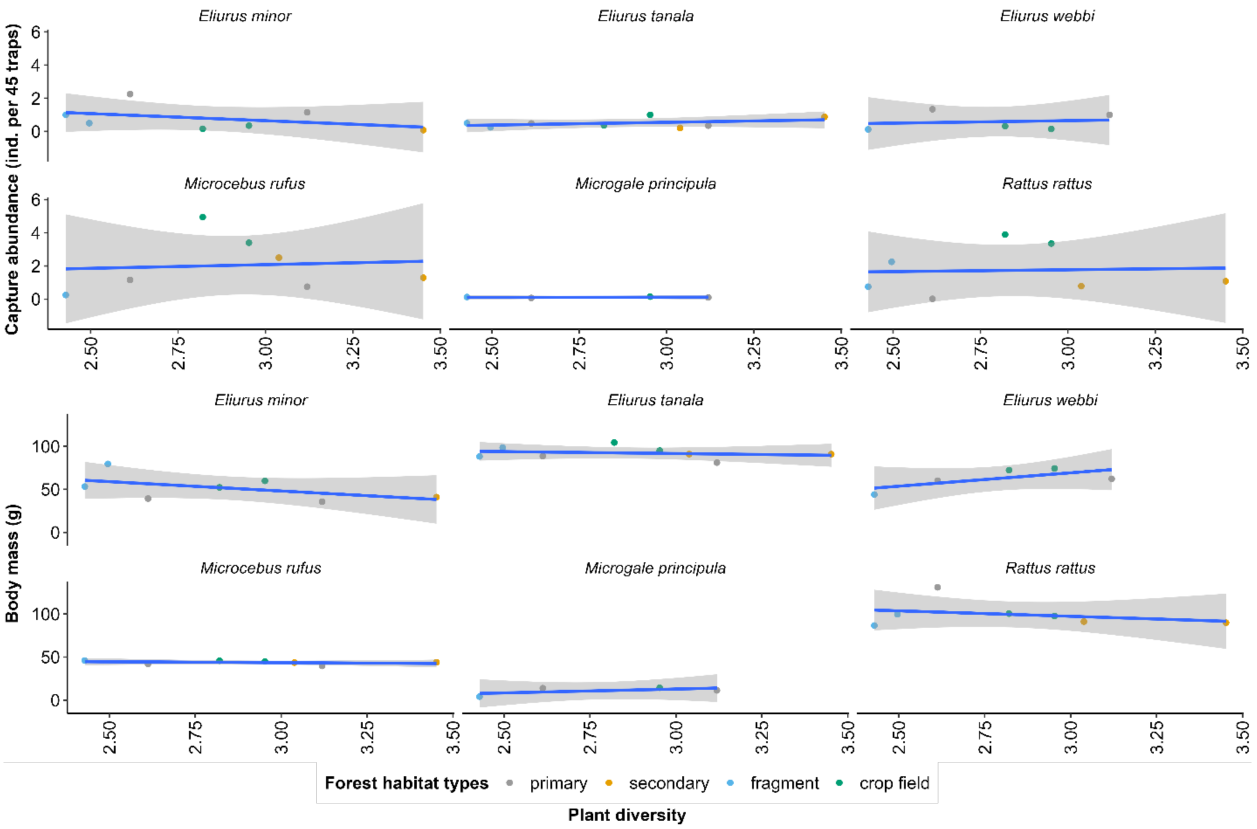


| Species | Capture abundance | | | Body mass | | |
|---|---|---|---|---|---|---|
| | $\chi^2$ | F | p | $\chi^2$ | F | P |
| ***Eliurus minor*** | 4.904 | 14.154 | < 0.001 | 6192.200 | 43.483 | < 0.001 |
| ***Eliurus tanala*** | 1.198 | 48.280 | < 0.001 | 41.770 | 1.731 | 0.189 |
| ***Eliurus webbi*** | 0.451 | 3.632 | 0.057 | 4108.900 | 70.175 | < 0.001 |
| ***Microcebus rufus*** | 55.809 | 43.409 | < 0.001 | 21.139 | 7.064 | < 0.01 |
| ***Microgale principula*** | 0.001 | 0.0169 | 0.681 | 394.86 | 79.421 | < 0.001 |
| ***Rattus rattus*** | 0.410 | 0.286 | 0.592 | 21.500 | 0.345 | 0.552 |

**Figure S3.** Changes in the capture abundance of small mammals within and around Ranomafana National Park in response to the variations of plant diversity with stepwise LMM coefficients: $\chi^2$ = sum of squares, F = model deviation from null model, and p = probability of deviation.

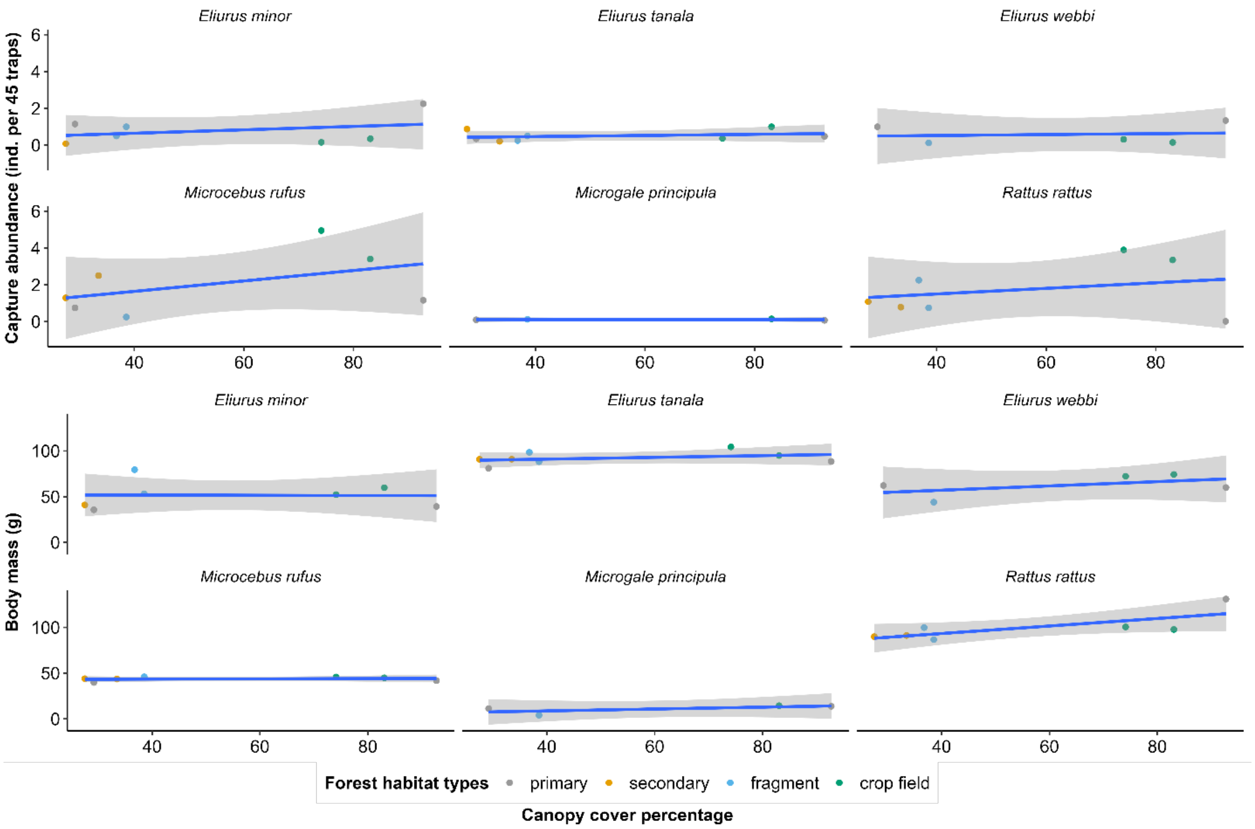


| Species | Capture abundance | | | Body mass | | |
|---|---|---|---|---|---|---|
| | $\chi^2$ | F | p | $\chi^2$ | F | P |
| ***Eliurus minor*** | 12.507 | 36.099 | < 0.001 | 3136.600 | 22.026 | < 0.001 |
| ***Eliurus tanala*** | 6.209 | 250.205 | < 0.001 | 32.980 | 1.364 | 0.244 |
| ***Eliurus webbi*** | 0.070 | 0.568 | 0.452 | 9407.700 | 160.673 | < 0.001 |
| ***Microcebus rufus*** | 41.744 | 32.469 | < 0.001 | 3.242 | 1.083 | 0.299 |
| ***Microgale principula*** | 0.030 | 260.328 | < 0.001 | 1594.81 | 320.772 | < 0.001 |
| ***Rattus rattus*** | 52.540 | 37.294 | < 0.001 | 24334.1 | 399.329 | < 0.001 |

**Figure S4.** Changes in the capture abundance of small mammals within and around Ranomafana National Park in response to the variations of forest canopy cover percentage with stepwise LMM coefficients: $\chi^2$ = sum of squares, F = model deviation from null model, and p = probability of deviation.

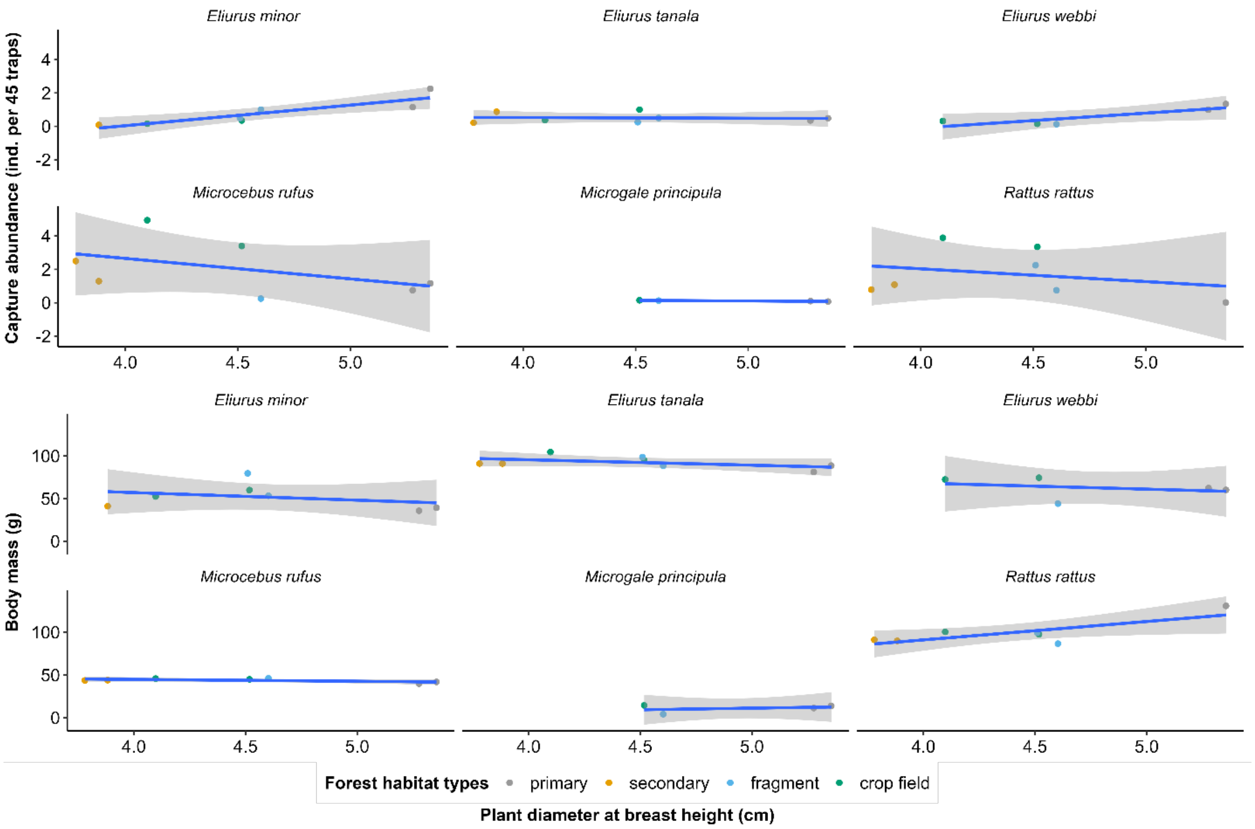


| Species | Capture abundance | | | Body mass | | |
|---|---|---|---|---|---|---|
| | $\chi^2$ | F | p | $\chi^2$ | F | P |
| ***Eliurus minor*** | 1.485 | 4.287 | < 0.05 | 8.200 | 0.057 | 0.810 |
| ***Eliurus tanala*** | 0.121 | 4.865 | < 0.05 | 637.540 | 26.346 | < 0.001 |
| ***Eliurus webbi*** | 2.970 | 23.653 | < 0.001 | 20.100 | 0.345 | 0.557 |
| ***Microcebus rufus*** | 10.374 | 8.069 | < 0.01 | 21.207 | 7.088 | < 0.01 |
| ***Microgale principula*** | 0.001 | 0.811 | 0.370 | 25.470 | 5.124 | < 0.05 |
| ***Rattus rattus*** | 35.150 | 24.951 | < 0.001 | 131.900 | 2.171 | 0.141 |

**Figure S5.** Changes in the capture abundance of small mammals within and around Ranomafana National Park in response to the variations of plant mean diameter at breast height (DBH) with stepwise LMM coefficients: $\chi^2$ = sum of squares, F = model deviation from null model, and p = probability of deviation.

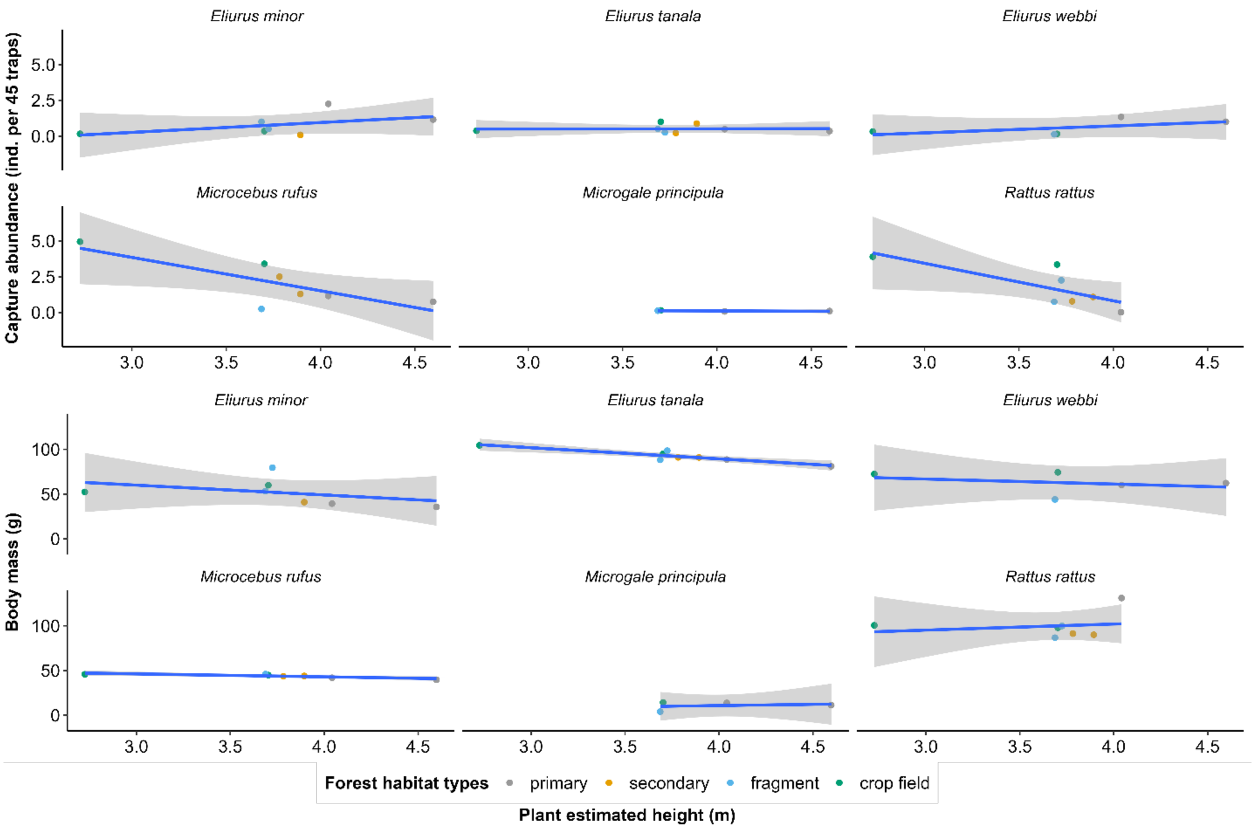


| Species | Capture abundance | | | Body mass | | |
|---|---|---|---|---|---|---|
| | $\chi^2$ | F | p | $\chi^2$ | F | p |
| ***Eliurus minor*** | 17.355 | 50.090 | < 0.001 | 2861.900 | 20.097 | < 0.001 |
| ***Eliurus tanala*** | 0.009 | 0.370 | 0.543 | 3029.300 | 125.185 | < 0.001 |
| ***Eliurus webbi*** | 14.870 | 118.412 | < 0.001 | 142.800 | 2.453 | 0.119 |
| ***Microcebus rufus*** | 69.412 | 53.990 | < 0.001 | 263.482 | 88.058 | < 0.001 |
| ***Microgale principula*** | 0.001 | 9.978 | < 0.01 | 171.200 | 34.434 | < 0.001 |
| ***Rattus rattus*** | 94.929 | 67.381 | < 0.001 | 4754.300 | 78.019 | < 0.001 |

**Figure S6.** Changes in the capture abundance of small mammals within and around Ranomafana National Park in response to the variations of mean plant estimated height with stepwise LMM coefficients: $\chi^2$ = sum of squares, F = model deviation from null model, and p = probability of deviation.
